# Extremely low arylsulfatase A enzyme activity does not necessarily cause symptoms: A long‐term follow‐up and review of the literature

**DOI:** 10.1002/jmd2.12293

**Published:** 2022-05-04

**Authors:** Lucia Laugwitz, Vidiyaah Santhanakumaran, Mareike Spieker, Judith Boehringer, Benjamin Bender, Volkmar Gieselmann, Stefanie Beck‐Woedl, Gernot Bruchelt, Klaus Harzer, Ingeborg Kraegeloh‐Mann, Samuel Groeschel

**Affiliations:** ^1^ Institute of Medical Genetics and Applied Genomics University of Tübingen Tübingen Germany; ^2^ Department of Neuropediatrics, Developmental Neurology and Social Paediatrics University of Tübingen Tübingen Germany; ^3^ Diagnostic and Interventional Neuroradiology Radiologic Clinics, University of Tübingen Tübingen Germany; ^4^ Institute of Biochemistry and Molecular Biology University of Bonn Bonn Germany

**Keywords:** *ARSA*, arylsulfatase A, MLD, MRI, newborn screening, ARSA pseudodeficiency

## Abstract

Metachromatic leukodystrophy (MLD) is an autosomal recessive lysosomal storage disease caused by deficiency of arylsulfatase A (ARSA). Heterozygous carriers of disease‐causing variants and individuals harbouring pseudodeficiency alleles in the *ARSA* gene exhibit reduced ARSA activity. In the context of these genotypes, low ARSA activity has been suggested to lead to an atypical form of MLD or other neurological abnormalities, but data are limited. The aim of our study was to analyse the impact of low ARSA activity in two subjects who are heterozygous for the ARSA pseudodeficiency allele and a disease‐causing variant. Biochemical testing included ARSA activity measurements and urinary sulfatide analysis. Biochemical data of a large cohort of MLD patients, heterozygotes, pseudodeficient individuals and healthy controls were analysed. MRI was performed at 3T using T1‐ and T2‐weighted sequences and MR spectroscopy. We present two long‐term follow‐ups who are heterozygous for the *ARSA* pseudodeficiency allele and a disease‐causing variant in the *ARSA* gene in cis. The two related index cases exhibit markedly reduced ARSA activity compared to controls and heterozygous carriers. The neurological evaluation and MRI do not reveal any abnormalities. Our data underline that extremely low enzyme activity due to a pseudodeficiency allele and a disease‐causing variant in the *ARSA* gene even in *cis* does not lead to clinical symptoms or pre‐symptomatic MRI changes suspicious for MLD. The review of literature corroborates that any association of low ARSA activity with disease features remains questionable. It seems important to combine the measurement of ARSA activity with elevated sulfatide as well as genetic testing, as done in current newborn screening approaches. Heterozygosity for metachromatic leukodystrophy and an arylsulfatase A pseudodeficiency allele does not cause neurological or neuropsychiatric features.

## 1 INTRODUCTION

Metachromatic leukodystrophy (MLD) is an autosomal recessive lysosomal storage disease caused by deficiency of arylsulfatase A (ARSA). ARSA deficiency leads to an accumulation of sulfatide substrates predominantly in the central nervous system and peripheral nervous system. Clinically, three different forms have been classified based on the age of onset: late infantile, juvenile and adult. To date, at least 280 biallelic missense and loss‐of‐function (LoF) variants have been disclosed in the *ARSA* gene (NM_000487.6) (wildtype: *ARSA* ^+^/*ARSA* ^+^) as disease‐causing according to the Human Genomic Mutation Database (HGMD) (09/2021). In addition to various disease‐causing variants (*ARSA* ^−^), non‐disease‐causing ARSA variants were long known (^1^, ^2^, ^3^) and later attributed to the polymorphisms referred to as the arylsulfatase A pseudodeficiency allele (*ARSA* ^*PD*^ *)*; (^1^, ^2^, ^3^, ^4^, ^5^, ^6^, ^7^). The term *pseudo*deficiency (*PD*) is misleading, as both heterozygous (*ARSA* ^*PD*^/*ARSA* ^+^) and homozygous carriers (*ARSA* ^*PD*^/*ARSA* ^*PD*^) exhibit a verifiable enzyme deficiency that accounts for a decreased amount of enzyme.^4^, ^5^, ^8^ The *PD* allele can bear two polymorphisms, c.*96A>G and c.1055A>G, p.(Asn352Ser), respectively, on the same allele.^9^ Referring to evolutionary studies, the hypothesis was raised that the variant c.*96A>G (allele frequency 0.04556 according to gnomAD) emerged on the background of the more ancient c.1055A>G polymorphism (allele frequency 0.1665 according to gnomAD) explaining their tightly linked localization.^10^, ^11^ The c.*96A>G polymorphism leads to the loss of the first polyadenylation signal downstream of the stop codon and results in a reduced translation of ARSA. The c.1055A>G, p.(Asn352Ser) polymorphism may occur isolated and results in the loss of one of three N‐glycosylation sites, causing a reduced half‐life of the enzyme. Studies examining the quantitative impact of each of the polymorphisms to activity reduction yielded different results; however, it seems that c.*96A>G contributes to the biochemical phenotype more than the c.1055A>G polymorphism.^9^, ^12^, ^13^ Although there is consensus that *PD* alleles have no clinical consequences in mono‐ and biallelic states,^7^, ^14^, ^15^ the biochemical signature deciphers enzyme deficiency levels that overlap with those of MLD patients.^9^, ^16^, ^17^ Individuals who carry both one *PD* allele and one disease‐causing variant in cis or in trans (*ARSA* ^*PD*^, *ARSA* ^−^/*ARSA* ^+^ or *ARSA* ^*PD*^/*ARSA* ^−^) show even lower ARSA activity levels than those who are homozygous for the *PD* allele (*ARSA* ^*PD*^/*ARSA* ^*PD*^ *)*, but remain clinically asymptomatic.^7^, ^17^ Despite a substantial reduction down to 5%–15% of normal, the enzyme activity remains sufficient to sustain an effectual sulfatide metabolism, as sulfatide accumulation or pathological urine excretion is not reported.^3^, ^17^, ^18^

The discrepancy between biochemical findings and clinical presentation is challenging in diagnosing and counselling patients and their families and becomes even more relevant as newborn screening is currently implemented.^19^, ^20^ At the same time, there are reports in the literature since the 1980s that individuals with low ARSA activity due to a *PD* allele and/or a disease causing *ARSA* variant might exhibit variable neurological or neuropsychiatric features (^14^, ^15^, ^21^, ^22^, ^23^, ^24^, ^25^, ^26^, ^27^).

Here, we present the first long‐term follow‐up of two related individuals with extremely low ARSA activity harbouring both *PD* polymorphisms and a disease‐causing variant in the *ARSA* gene in cis (*ARSA* ^*PD*^, *ARSA* ^−^/*ARSA* ^+^). We provide statistical analyses comparing biochemical data of a large cohort of MLD patients with heterozygotes, pseudodeficient individuals and controls. Moreover, we critically discuss cases and cohort studies with low ARSA activity presented in the literature.

## 2 METHODS

ARSA activity was measured as described by Strobel et al. in white blood cell lysates using p‐nitrocatecholsulfate as substrate. After a 48‐h incubation period at 8°C, the reaction was stopped with NaOH and the absorption was measured at 514 nm.^28^, ^29^, ^30^ Urine sulfatide analysis: The qualitative analysis of sulfatide excretion was performed by two‐dimensional thin‐layer chromatography (TLC) according to our laboratory standard procedures.^31^, ^32^, ^33^

Statistical analyses were conducted using Graph Pad Prism Software, Version 9. Means were compared using Mann–Whitney test.

Sanger sequencing was performed. Genomic DNA was extracted from blood collected in EDTA vials using the Flexi Gene DNA Kit (Qiagen) according to the manufacturer's instructions. PCR was performed on a Perkin Elmer Gene Amp PCR System 9700 cycler.

MRI was acquired on a 3T Siemens Skyra using a previously described protocol for white matter pathology^34^ including T1‐ and T2‐weighted sequences and MR spectroscopy at short (TE 40 ms) and long (TE 135 ms) echo times.

## 3 CASE PRESENTATION

**FIGURE 1:**
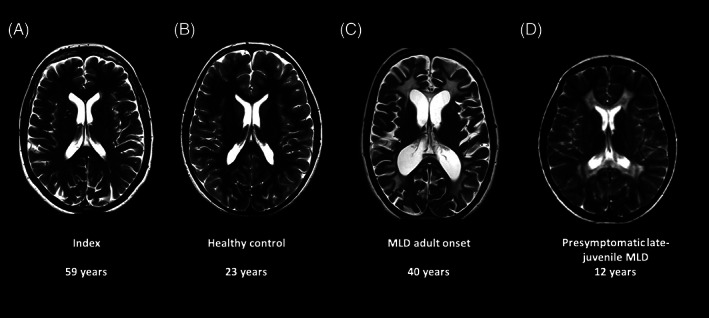
MRI data. Cerebral T2‐weighted MRI of the index case at the age of 59 years (A) with no signs of demyelination, which is comparable to that of a healthy adult control (B). In contrast, T2‐weighted MRI of a patient with adult onset of MLD (C) and juvenile onset of MLD (D) reveal marked demyelination

We report on the long‐term follow‐up of a 61‐year‐old healthy woman (index 1). Following her niece's diagnosis of juvenile MLD, she was biochemically and genetically tested for MLD in her 30s. She was identified as a carrier of the c.1055A>G and c.*96A>G *PD* polymorphisms as well as the disease‐causing c.1283C>T, p.(Pro428Leu) (allele frequency 3.83 × 10^−5^ according to gnomAD) variant on the same allele. Intriguingly, her ARSA enzyme activity was remarkably low. She was reportedly healthy throughout her life. Birth history and childhood were unremarkable, developmental milestones were reached at the expected ages. She was especially dexterous regarding fine and gross motor skills. She achieved a university degree in mechanical engineering and worked as a technical illustrator since then. She is a mother of four clinically healthy children. One of her healthy daughters was identified to harbour the same genotype at the age of 37 years (index 2). The thorough clinical examination of index 1 showed an unremarkable neurological status regarding mental status, gross and fine motor functions, cranial nerves, deep tendon reflexes, sensory system and coordination at the age of 61 years. A cerebral MRI was last acquired for this study and rated as unremarkable, with normal white matter signal of T1‐ and T2‐weighted and normal MR spectroscopy, without increased choline, inositol, or lactate, and with normal NAA level (Figure 1A).

**FIGURE 2:**
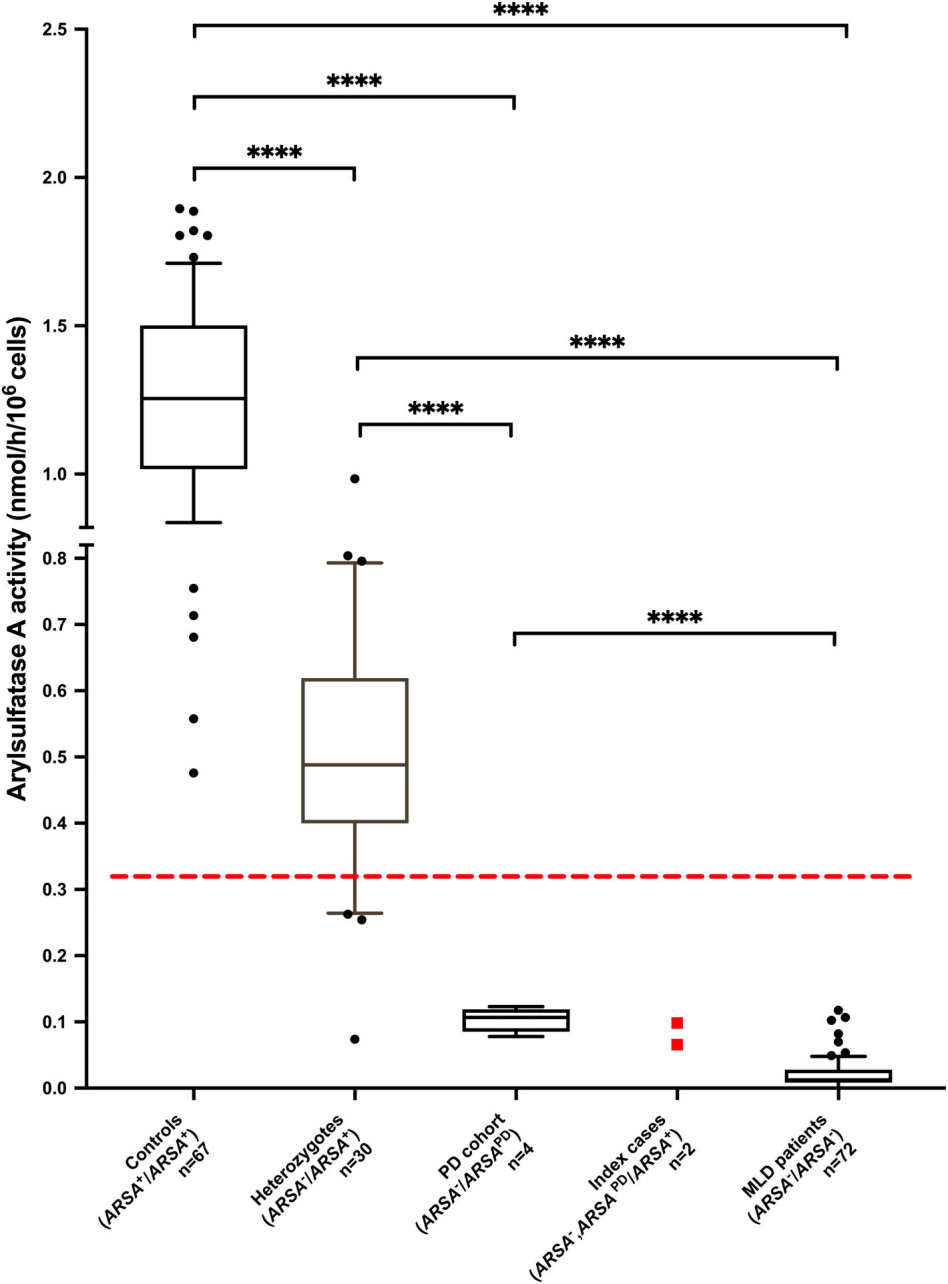
Arylsulfatase A (ARSA) enzyme activity in different cohorts. The ARSA activity is indicated as nmol/h/10^6^ cells. Lower limit, indicated by the dashed line, was set as 0.33 nmol/h/10^6^ cells (26% of mean control activity). The activity of the control cohort was 1.26 ± 0.33 nmol/h/10^6^cells (*n* = 67). With 1.6% of mean control activity, ARSA activities were significantly lower in MLD patients (mean 0.02 ± 0.02 nmol/h/10^6^cells; *n* = 72 [*p* < 0.0001]). Heterozygotes show 40.2% of mean control activity (mean 0.51 ± 0.19 nmol/h/10^6^cells; *n* = 30). The index case 1 revealed an enzyme activity of 7.8% (0.098 nmol/h/10^6^cells) (labelled in red) and index 2 5.2% (0.066 nmol/h/10^6^cells) (labelled in red) compared to mean control activity, which is in the range of the *PD* cohort (mean 0.10 ± 0.02 nmol/h/10^6^cells; *n* = 4). All cohorts differed significantly based on Mann–Whitney test (*p* < 0.0001). Whiskers at 10–90 percentiles

The ARSA activities measured in leukocytes of both index cases were compared to our in‐house cohort (Figure 2). Within this cohort, the mean values of the ARSA activity in heterozygous carriers (*ARSA* ^−^/*ARSA* ^+^) were significantly lower than those of control individuals (wildtype: *ARSA* ^+^/*ARSA* ^+^) and significantly higher than the mean values of confirmed MLD patients. The indices' ARSA values are clearly below the set lower limit (0.33 nmol/h/10^6^cells), but above the MLD cohort (mean 0.02 ± 0.02 nmol/h/10^6^cells; *n* = 72). Compared to mean control values, the residual ARSA activity was 7.8% (0.098 nmol/h/10^6^cells) for index 1 and 5.2% (0.066 nmol/h/10^6^cells) for index 2 comparable to ARSA activity values found in the cohort of individuals harbouring a pseudodeficiency allele (*PD* cohort) (mean 0.10 ± 0.02 nmol/h/10^6^cells; *n* = 4). In this *PD* cohort ARSA values are significantly reduced compared to that of the controls (*ARSA* ^+^/*ARSA* ^+^) (mean 1.26 ± 0.33 nmol/h/10^6^cells; *n* = 67) and heterozygous carriers (*ARSA* ^−^/*ARSA* ^+^) (mean 0.51 ± 0.19 nmol/h/10^6^cells; *n* = 30) (Figure 2). The high standard deviation in the heterozygous cohort including outliers with very low ARSA activity can partially be explained through heterozygous carriers of loss‐of‐function (LOF) variants. Only the index cases carried the additional disease causing *ARSA* variant in cis (*ARSA* ^*PD*^, *ARSA* ^−^/*ARSA* ^+^). For all individuals of the PD cohort, compound heterozygosity was confirmed (*ARSA* ^PD^/*ARSA* ^−^). In contrast to the cohort of MLD patients, an elevated excretion of sulfatides in urine was not detectable by the routine diagnostic assay in the index cases nor compound heterozygous carriers.

## 4 DISCUSSION

Here, we present the long‐term follow‐up of two related individuals harbouring both a disease‐causing variant in the *ARSA* geneand both *PD* polymorphisms in cis. This combination has caused a remarkably low residual ARSA activity in these individuals and is clearly below the activity of controls and heterozygous individuals (Figure 2). At the same time, these individuals do not show increased sulfatide excretion and do not exhibit symptoms or MRI changes for MLD.

While these *PD* polymorphisms lead to a reduced translation and/or a reduced half‐life of the ARSA enzyme, the hypothesis has been proposed that the residual enzyme activity remains sufficient to sustain an effectual sulfatide metabolism.^5^, ^35^ Conzelmann and Sandhoff suggest a critical threshold of residual enzyme activity, below which the substrate influx rate exceeds the degradation rate. Subsequently, sulfatides accumulate and the lysosomal storage disease will manifest over time.^36^ The reduced ARSA activity due to *PD* polymorphisms and/or heterozygous disease‐causing *ARSA* variants seems to be above this critical threshold. Hence, pathological sulfatide accumulation is not reported.^3^, ^17^, ^18^

Comparing ARSA activity between the different genetic cohorts (Figure 2), our results show that compound heterozygotes for a disease‐causing variant in the *ARSA* gene and an ARSA pseudodeficiency allele (*ARSA* ^−^/*ARSA* ^PD^) exhibit only about 5%–10% of the mean ARSA activity of the normal controls. Comparing these low levels to the residual ARSA activity of about 13% reported for white blood cells of *PD* homozygotes (for both polymorphisms),^37^ the difference may be explained by an additional loss of ARSA activity when one of the two *PD* alleles is replaced by an allele with a disease‐causing variant. However, the finding in some MLD patients (Figure 2) that even biallelic pathogenic variants can lead to similar residual ARSA activities as in MLD heterozygotes with additional *PD* polymorphisms in cis or in trans is remarkable. The fact that the index 1 and index 2 show only about 5%–8% of residual ARSA activity despite having an intact second allele remains difficult to explain. We consider the possibility that the product of the first allele, a non‐functional ARSA protein, might interact with the remaining ARSA due to the intact second allele, inhibiting parts of the enzyme activity. Such an interaction might be favoured by the loss of proline 428 in the non‐functional ARSA protein, which alters this protein‘s secondary structure and folding. The altered protein might act by steric hindrance on the residual ARSA. Another possibility could be that the non‐functional ARSA protein was overproduced in response to the instability conferred on it by the *PD* variant c.1055A>G.^38^ The resulting high amounts of non‐functional protein might again inhibit the residual ARSA. However, these considerations require experimental confirmation. To the best of our knowledge, the finding of residual ARSA activities lower than 10% of normal controls in the two index patients bearing the two *PD* variants together with a pathogenic variant on one *ARSA* allele, the other allele obviously being wildtype, so far is unique. An *ARSA* allele with the two *PD* variants and a pathogenic variant in cis seems to have an unexplained potential to suppress at least in vitro the ARSA activity produced by a concomitant wildtype allele.

It is important to note that while the ARSA activity in the *PD* cohort was on average higher than in MLD patients, there was considerable overlap, which corroborates previous findings.^17^ In contrast to Lugowska and colleagues, the ARSA/β‐galactosidase ratios did not improve the discriminatory power (data not shown). Subsequent urinary sulfatide testing is an important step in the diagnostic procedure of suspected MLD cases with ARSA activity below set thresholds, as this method can reliably distinguish the MLD and *PD* cohort: the sulfatide excretion is only minimal or even undetectable in the heterozygous or pseudodeficient individuals.^1^, ^2^, ^3^, ^17^

The discussion on the implication of low ARSA activity was initiated by case reports on healthy relatives of MLD patients.^1^, ^2^, ^3^, ^39^. Although full clinical, genetic or neuroimaging work‐up was not done, these single reports suggested no disease‐causing impact of low ARSA activity due to *ARSA* pseudodeficiency alleles.^1^, ^2^ Penzien and colleagues investigated a cohort of 16 individuals that were heterozygous for a *PD* allele in trans with disease‐causing variants in the *ARSA* gene (*ARSA* ^*PD*^/*ARSA* ^−^), among which 4 individuals showed neuroradiological and/or clinical anomalies. During a clinical follow‐up period of up to 11 years, symptoms neither showed progression nor resemblance to any typical MLD features. Thus, the authors concluded that the association was not causal^7^ (Table 1).

**TABLE 1:** Review of literature on clinical effects of low ARSA activity

Publication	PMID	Type	Study cohort and clinical features	Genotype	Sulfatide excretion	Conclusion
Lott et al. 1976	8599	CR	Healthy individuals (*n* = 2)	n.t.	no	Low ARSA activity due to putative PD alleles and/or *ARSA* ^−^/*ARSA* ^+^ does not cause disease features
Dubois et al. 1977	15 452	CR	Healthy relatives of MLD patient (*n* = 4)	n.t.	n.t.	Low ARSA activity in putative heterozygotes does not cause disease features
Berger et al. 1999	9 888 390	CR	Healthy relatives of MLD patient (*n* = 3)	*ARSA* ^−^/*ARSA* ^+^	minimal	Low ARSA activity due to a LoF variant does not cause disease features
Penzien et al. 1993	8 095 368	CS	*ARSA* ^PD^/*ARSA* ^−^ compound heterozygotes (*n* = 16) with low ARSA activity; among which 2 cases with unspecific neurological symptoms, but normal MRI, 2 healthy cases with non‐MLD like MRI changes	*ARSA* ^PD^/*ARSA* ^−^ or putative *ARSA* ^PD^/*ARSA* ^−^ (*n* = 10)	minimal (4/9); no (3/9)	Neurological symptoms are unrelated to *ARSA* ^PD^/*ARSA* ^−^ genotype and low ARSA activity; compound heterozygotes are not at an increased risk for developing progressive nervous system disease
Tylki‐Szymanska et al. 2002	12 116 203	CS	Hyperreflexia: *ARSA* ^−^/*ARSA* ^+^(10/21); *ARSA* ^−^/*ARSA* ^*PD*^(3/5); controls (2/19); MOBD: *ARSA* ^−^/*ARSA* ^+^ and *ARSA* ^−^/*ARSA* ^*PD*^(7/26); controls (1/19)	*ARSA* ^−^/*ARSA* ^+^(*n* = 21); *ARSA* ^PD^/*ARSA* ^−^(*n* = 3); *ARSA* ^−^, *ARSA* ^*PD*^/*ARSA* ^+^(*n* = 2); *ARSA* ^−^, *ARSA* ^*PD*^/*ARSA* ^*PD*^(*n* = 1)	minimal	Higher incidence of non‐progressive MOBD in *ARSA* ^−^/*ARSA* ^+^ and *ARSA* ^PD^/*ARSA* ^−^ genotypes than in controls
Butterworth et al. 1978	699 360	CR	Index with very low ARSA activity and neurometabolic disorder, but no signs of MLD, (*n* = 1); healthy relatives with low ARSA activity (*n* = 3)	n.t. index: putative *ARSA* ^PD^/*ARSA* ^−^	n.t.	Extremely low ARSA activity due to a putative *ARSA* ^PD^/*ARSA* ^−^ genotype as coincidental with another, neurometabolic disorder
Danesiono et al. 1984	6 149 830	CR	Index (non‐progressive DD, hepatomegaly) with very low ARSA activity (*n* = 1); healthy parents with moderately reduced ARSA activity (*n* = 2)	n.t.	n.t.	Extremely low ARSA activity in a patient with suspected neurogenetic disorder as coincidental finding
Hohenschutz et al. 1988	2 906 225	CR	Index with low ARSA activity and encephalomyelitis disseminata (*n* = 1)	*ARSA* ^PD^/*ARSA* ^−^	minimal	Possible association of low ARSA activity and sulfatide excretion with neuropsychiatric symptoms and demyelination
Grasso et al. 1989	2 572 149	CR	2 siblings with low ARSA activity and myoclonic epilepsy (*n* = 2)	n.t.	n.t.	Case report of neurological patients (non‐MLD) with low ARSA activity
Tinuper et al. 1994	7 908 874	CR	Index with low ARSA activity and myoclonic epilepsy (*n* = 1)	*ARSA* ^PD^/*ARSA* ^PD^	n.t.	Possible association of low ARSA activity with myoclonic epilepsy
Sangiorgi et al. 1991	1 683 156	CS	Paediatric epilepsy patients and (*n* = 93); children with DD (*n* = 47); controls (*n* = 71)	n.t.	no	Possible association with neuropediatric disorders as 24% of epileptic patients and 30% of patients with DD exhibit low ARSA activity (versus 1.4% of controls)
Kappler et al. 1991	1 687 779	CS	MS patients (*n* = 160); controls (*n* = 160)	Putative *ARSA* ^PD^/*ARSA* ^PD^: MS (4/160); controls (1/160)	n.t,	Higher incidence of putative *ARSA* ^PD^/*ARSA* ^PD^ genotype (4/160) and low ARSA activity (5/160) in the MS cohort compared to controls (1/160 and 2/160)
Goldenfum et al. 1993	7 907 382	CS	Patients with neurological symptoms (*n* = 28)	patients: *ARSA* ^PD^/*ARSA* ^PD^ (*n* = 1); *ARSA* ^PD^/*ARSA* ^+^ (*n* = 16); healthy relatives: *ARSA* ^PD^/*ARSA* ^+^ (*n* = 3); *ARSA* ^PD^/*ARSA* ^−^ (*n* = 1)	n.t.	Extremely low ARSA activity due to *ARSA* ^PD^/*ARSA* ^−^, low ARSA activity due to *ARSA* ^PD^/*ARSA* ^PD^ and *ARSA* ^PD^/*ARSA* ^+^ genotypes; no causal association of neurological features and low ARSA activity
Mahon‐Haft et al. 1981	6 117 201	CS	Adult psychiatric patients with schizophrenia (*n* = 18); controls (*n* = 6)	n.t.	minimal (3/18)	3/18 patients with low ARSA and sulfatide excretion suggested as adult MLD cases; 1/18 case with low ARSA activity without sulfatide excretion suggested as putative PD case
Herska et al. 1987	2 882 680	CS	Adult psychiatric patients with schizophrenia or manic depression (*n* = 295)	n.t.	n.t.	No statistical association of low ARSA activity and psychiatric disorders (<1% of patients with low ARSA activity)
Propping et al. 1986	2 877 931	CS	Adult psychiatric patients (*n* = 2107)	n.t.	n.t.	Moderately reduced ARSA activity in 15% of acute psychiatric and 10% of chronic inpatients versus 11% in controls
Hohenschutz et al. 1989	2 565 866	CS	Adult psychiatric patients (*n* = 14); controls (*n* = 7) (identified with low ARSA activity in^25^)	putative *ARSA* ^PD^/*ARSA* ^PD^ (*n* = 14) putative *ARSA* ^PD^/*ARSA* ^+^ (*n* = 6) putative *ARSA* ^PD^/*ARSA* ^−^ (*n* = 1)	n.t.	Higher incidence low ARSA activity due to the *ARSA* ^PD^/*ARSA* ^PD^ or *ARSA* ^−^,*ARSA* ^PD^/*ARSA* ^+^ genotype in psychiatric patients
Hulyalkar et al. 1984	6 146 271	CS	Adult psychiatric patients with schizophrenia (*n* = 100); non‐schizophrenic psychiatric disorders (*n* = 100) and/or alcoholism (*n* = 56); controls (*n* = 100)	n.t.	n.t.	Possible association of low ARSA activity and alcoholism, no differences comparing other disease groups
Heavey et al. 1990	1 975 970	CS	Psychotic disorders (*n* = 45); controls (*n* = 30); patients with neurological symptoms (*n* = 58); putative *ARSA* ^−^/*ARSA* ^+^ (*n* = 22)	n.t.	n.t.	Possible association of slightly reduced ARSA activity and schizoaffective psychosis (3/45) comparing mean values among cohorts
Shah et al. 1995	2 856 894	CS	Psychiatric and/or neurological patients (*n* = 149); controls (*n* = 30)	n.t.	no	Possible association of low ARSA activity with psychiatric disorders (39/14 patients versus 1/30 controls)

Abbreviations: CS, Cohort study; CR, case report; DD, developmental delay; LoF, loss of function; MOBD, micro‐organic brain damage; MS, multiple sclerosis; n.t, not tested; PD, pseudodeficiency.

Interestingly, Tylki‐Szymanska and colleagues, who investigated a cohort of heterozygotes (*ARSA* ^−^/*ARSA* ^+^ or *ARSA* ^*PD*^/*ARSA* ^+^ or *ARSA* ^*PD*^,*ARSA* ^−^/*ARSA* ^+^) and compound heterozygotes (*ARSA* ^*PD*^/*ARSA* ^−^), postulated a slightly higher incidence of what they termed “micro‐organic brain damage” (MOBD).^27^ Symptoms of MOBD were assessed by neurological and psychometric examinations. Besides hyper‐reflexia being prominent in female carriers, neurological examinations did not differ generally from controls. During IQ testing, no distinction was seen between the tested cohort and controls. Benton test to detect impairment of visual memory revealed 7/25 cases in the study cohort versus 1/19 case among controls. Thus, the final conclusion of a higher incidence of MOBD in heterozygotes (*ARSA* ^−^/*ARSA* ^+^ or *ARSA* ^*PD*^/*ARSA* ^+^ or *ARSA* ^*PD*^, *ARSA* ^−^/*ARSA* ^+^) and compound heterozygotes (*ARSA* ^*PD*^/*ARSA* ^−^) was strongly based on results of Benton test only, with limited statistical validity and the results remain somewhat inconclusive.

Moreover, in sporadic case reports on patients with undefined neuropediatric or neurometabolic disorders, the low ARSA activity was most likely coincidental and the reported clinical features were clearly different from MLD‐like disease features.^40^, ^41^ The controversy was fuelled by a case report on a patient with *encephalitis disseminata*, who was found to be compound heterozygous for a *PD* allele and a disease‐causing variant in the *ARSA* gene (*ARSA* ^*PD*^/*ARSA* ^−^).^23^ Although the authors suggest that low ARSA activity might have caused a long‐term accumulation of sulfatides and thus facilitated the demyelinating process and neuropsychiatric symptoms, up to date, no further pathophysiological, neuroradiological or statistical evidence supports this hypothesis.^22^, ^23^ In addition, other studies have suggested an association with myoclonic seizures and low ARSA activity.^42^, ^43^ Sporadic case reports associating ARSA deficiency with otherwise unexplained neurological disorders must be interpreted with caution, as hereditary disorders were broadly underdiagnosed before the introduction of next‐generation sequencing (NGS) techniques into routine diagnostics. In a cohort of 140 paediatric patients with varying neurological features, Sangiori and colleagues identified more individuals with low or extremely low ARSA activity but without urinary sulfatide excretion among patients with epilepsy (25%) or psychomotor delay (30%) than among controls (1.4%).^26^ Unfortunately, neuroradiological and genetic evaluation of these individuals was not provided. A comparable study by Goldenfum and colleagues in an adult cohort with variable neurological symptoms (*n* = 28) suggested, however, no causal association of low ARSA activity and neurological disorders.^16^

In addition to speculation that the biochemical phenotype of reduced ARSA activity leads to an atypical form of MLD, some studies discuss the contribution of low ARSA activity to other multifactorial disorders. Kappler and colleagues suggest an association of low ARSA activity and multiple sclerosis (MS) in a cohort of 160 patients, as four individuals in the MS cohort versus only one individual among the controls were found to be pseudodeficient.^14^, ^15^

However, a causal pathomechanism has not yet been established to support these hypotheses, and larger well‐powered studies are needed to statistically validate an association.

Several authors have initiated a discussion on low ARSA activity as a contributing factor to psychiatric disorders or even an atypical MLD disease courses.^18^, ^21^, ^24^, ^25^, ^45^, ^46^ However, the correlation in these screening studies in rather small cohorts was not statistically convincing in comparison to control cohorts and might be explained by the high prevalence of *PD* alleles and/or heterozygous disease‐causing variants in *ARSA* as pointed out by Herska and colleagues.^44^ Moreover, most cohort studies lack detailed genetic data as well as functional testing for sulfatide excretion; hence, adult MLD cases were not excluded systematically and MRI data were not provided.^21^, ^24^, ^25^, ^45^ To date, the hypothesis that variably reduced ARSA activity in vitro imposes a risk factor for a very heterogeneous spectrum of psychiatric disorders due to an alleged accumulation of sulfatides has not been corroborated by functional or statistical evidence.

Reviewing the literature rendered the challenge that some studies refer to individuals with low ARSA activity without additional sulfatide testing or genetic confirmation. Other studies do not specify the underlying *PD* alleles or allelic localization of the additional disease‐causing variant. However, these genetic details clearly impact the biochemical and clinical phenotype. Therefore, comparative studies in statistically meaningful cohorts are needed to provide further insights. Most importantly, besides considerate biochemical testing, a neuroradiological work‐up, NGS‐based genetic testing and a thorough clinical examination are required to rule out other aetiologies for any neurological and/or psychiatric phenotype in setting of a low ARSA activity. However, most studies and case reports lack such a comprehensive investigation.

### 4.1 Limitations

The genotype presented in these cases differs from *PD* cohorts usually described and, to the best of our knowledge, has not been discussed in literature. However, as both individuals in this case study are related, findings cannot be generalized and further studies in larger cohorts are warranted. Furthermore, no additional biochemical analysis by sulfatide loading test or quantitate sulfatide analysis as well as experimental work‐up on hypotheses regarding the structural and functional consequences of the underlying genotype were conducted. However, as the main intention was to discuss postulated clinical manifestation in subjects with extremely low ARSA activity the clinical work‐up in these cases was extensive and sufficient for the purpose of this study.

Current literature on this topic is scarce and majority of articles reviewed here arose in an era before NGS diagnostics. More recent studies have associated genetic dispositions like *PD* alleles or heterozygosity for pathogenic *ARSA* variants with various other disorders like cerebral palsy,^47^ Down Syndrome,^48^ dementia,^48^ Parkinson's disease^49^ or cardiovascular disorders.^50^ Unfortunately, these studies often provide no biochemical studies at all or refer to ARSA values with only minimal reduction in comparison to controls. None of these studies provides an experimental work‐up and again lack statistically meaningful cohort sizes for genome‐wide association studies. Taken together, our literature review revealed partly unresolved issues in older studies and a certain gap in current knowledge, which will become relevant again as newborn screening and NGS sequencing become widely available.

## 5 CONCLUSION

In conclusion, this comprehensive literature review, our data and the presented two long‐term follow‐ups decipher that the association of low ARSA activity and any neurological disease feature needs to be questioned critically, especially due to the high prevalence of heterozygous disease‐causing variants in the *ARSA* gene and *PD* alleles in the general population according to gnomAD,^51^ leading to reduced enzyme activity. In relatives of MLD patients, there is a considerable statistical probability of identifying members who harbour *PD* alleles in addition to the disease‐causing variant in *ARSA* and consequently exhibit very low ARSA activity. Considering the incredible burden that a diagnosis of MLD creates in potentially affected families, we call for the utmost diligence when counselling patients and their families, as isolated low ARSA activity does not necessarily cause neurological symptoms or a (mild) form of MLD. The additional investigation of sulfatides in urine in those individuals can sufficiently exclude MLD as done in specialized laboratories and pilot studies for newborn screening, ideally combined with genetic confirmation.^19^, ^20^ And most importantly, thorough diagnostic work‐up needs to incorporate detailed clinical and neuroimaging into the genetic and biochemical information.

## FUNDING INFORMATION

Open Access funding enabled and organized by Projekt DEAL. WOA Institution: N/A Consortia Name: Projekt DEAL. This work was supported by Deutsche Forschungsgesellschaft grant GR 4688/2‐1. The author(s) confirm(s) independence from the sponsors; the content of the article has not been influenced by the sponsors.

## CONFLICT OF INTEREST

The authors declare that they have no competing interests. Samuel Groeschel received institutional research support from Shire plc. He is advisor and coinvestigator for trials in MLD (Shire/Takeda, Orchard, Bioclinica), but receives no personal payment related to this role. Ingeborg Kraegeloh‐Mann received travel funds from Shire/Takeda. Outside the submitted work, Benjamin Bender is co‐founder and shareholder of AIRAmed GmbH.

## ETHICS STATEMENT

The work described here has been carried out in accordance with The Code of Ethics of the World Medical Association (Declaration of Helsinki) for experiments involving humans. A patient consent statement was obtained according to local standards (ethics number 948/2018BO2).

## PATIENT CONSENT

Informed consent was obtained for publication of data.

## INSTITUTIONAL COMMITTEE FOR CARE AND USE OF LABORATORY ANIMALS

Not applicable.

## Data Availability

Data and material are available upon request.
